# Immediate and Long-Term Effectiveness of a Therapeutic Exercise Protocol in Patients with Dementia

**DOI:** 10.3390/jcm15041482

**Published:** 2026-02-13

**Authors:** Ferreira-Sánchez María del Rosario, García-Macías Celia, Alarcón-Jiménez Jorge, Martín Jiménez Ana, Gómez-Sánchez Sonia, De Bernardo Nieves, Sánchez-Jiménez Elena

**Affiliations:** 1 Faculty of Health Sciences, Catholic University of Avila, C/Canteros S/N, 05005 Ávila, Spain; mrosario.ferreira@ucavila.es (F.-S.M.d.R.); celia.garcia@ucavila.es (G.-M.C.); ana.martin@ucavila.es (M.J.A.); sonia.gomez@ucavila.es (G.-S.S.); elena.sanchez@ucavila.es (S.-J.E.); 2 Department of Physiotherapy, Catholic University of Valencia, 46001 Valencia, Spain; nieves.debernardo@ucv.es

**Keywords:** therapeutic exercise, dementia, motor function, balance, gait

## Abstract

**Background/Objectives**: Therapeutic exercise (TE) has been shown to be an effective tool for slowing physical and cognitive decline in patients with dementia. However, its true impact on physical and functional variables, as well as the duration of its effects once therapy is discontinued, remains unclear. The aim was to analyze the short- and medium-term effects of a structured and monitored TE program on motor function in patients with dementia. **Methods**: A pre–post clinical trial was conducted in individuals with a medical diagnosis of mild-to-moderate cognitive impairment (Mini-Mental State Examination scores between 10 and 23) who had not engaged in regular exercise during the previous 6 months. The study variables and their measurement tools included general motor function (Short Physical Performance Battery), trunk control (Trunk Control Test), balance (Berg Balance Scale), overall mobility and gait (Timed Up and Go Test), and degree of independence in activities of daily living (ADLs) (Barthel Index). Participants completed a 12-week TE intervention at moderate intensity, 3 days per week for 45 min sessions. The program included aerobic training and strength, coordination, flexibility, and balance exercises. TE intensity was monitored through heart rate and dynamic maximal resistance. Assessments were conducted at baseline (t0), immediately after the program (t1), and 6 months after completion (t2). **Results**: Significant global longitudinal effects of time were observed for general motor function, balance, trunk control, and mobility and gait, whereas no significant global effect was detected for independence in activities of daily living. Post-intervention changes were non-significant; however, several pairwise comparisons showed moderate-to-large effect sizes. Follow-up assessments revealed shifts in performance distributions consistent with functional decline. **Conclusions**: A structured TE program performed at moderate intensity may help slow or attenuate the physical decline experienced by individuals with dementia.

## 1. Introduction

Dementia is a clinical syndrome characterized by an acquired and persistent deterioration of higher brain functions (memory, language, orientation, calculation, spatial perception, etc.), which leads to loss of patient autonomy and severe limitations in social, occupational, and leisure activities [1].

In 2021, 57 million people worldwide were living with dementia, with 10 million new cases diagnosed each year; Alzheimer’s disease (AD) accounts for 60–70% of these cases [2]. Age is the primary risk factor for AD and is even more determinant than genetic burden and family history [3,4]. Demographic studies estimate that the prevalence of dementia will rise to 82 million people by 2030 and to 152 million by 2050, with consequent social, economic, and healthcare implications worldwide [2,5,6]. Due to its chronic and incurable nature, the effectiveness of physical exercise has been investigated as a complementary non-pharmacological treatment, showing numerous benefits for the progression and prevention of dementia [7,8].

Therapeutic exercise (TE) has demonstrated a wide range of positive effects across nearly all domains (cognitive, physical, biochemical, functional, and neuropsychiatric) and is not associated with adverse effects, in contrast to pharmacological interventions [9,10]. Recent systematic reviews describe the benefits of physical exercise across all stages of the disease—from its preclinical phase to moderate and advanced stages—by exerting a direct influence on the disease’s pathophysiology [11,12,13].

Physical exercise triggers a cascade of physiological responses, including angiogenesis, neurogenesis, synaptogenesis, stimulation of neurotrophic factors, and improved endothelial function, all of which promote learning, memory, and neuroplasticity [14,15,16,17]. Comprehensive systematic reviews conclude that the biological mechanisms associated with exercise activate neurogenesis through both direct and indirect increases in BDNF production—mediated by the upregulation of cathepsin B and irisin—along with lactate accumulation in the hippocampus, which further enhances learning and memory, and the regulated production of ketone bodies [18,19,20,21].

Physical exercise also promotes regulation of the hypothalamic–pituitary–adrenal axis and increases brain volume [22]. Moreover, it significantly modulates oxidative stress, cortisol levels, and insulin and glucose signaling [11,23]. Oxidative stress is a hallmark of AD’s pathogenesis, and exercise contributes to reducing proinflammatory markers while enhancing the brain’s antioxidant capacity [21,24]. Physical exercise induces an anti-inflammatory shift in the hippocampus by increasing anti-inflammatory cytokines (IL-4β and IL-10β) and reducing proinflammatory cytokines such as TNF-α, IL-1β, and IL-6 [15,18].

Although physical exercise is frequently combined with other therapeutic approaches, exercise alone has been shown to reduce the risk of developing AD [25,26,27], improve independence in activities of daily living (ADLs) [28,29,30,31], delay loss of autonomy, enhance general physical capacity (balance and gait) [29,30,32,33], improve overall cognitive status (language and memory) [29,32,34], reduce neuropsychiatric symptoms (anxiety and depression) [30,32], increase hippocampal volume [35], and reduce overall healthcare expenditure [29].

Despite the well-established effects of physical and TE on disease progression and symptoms, it remains unclear whether structured protocolized exercise provides more benefits than non-standardized training and whether these effects persist after the discontinuation of a structured program. There is extensive evidence regarding the low adherence to treatment observed in patients with dementia and mild cognitive impairment (MCI) [25,28,36]. Although adherence has been studied mainly in relation to pharmacological treatment, research indicates that cognitive impairment is associated with poorer adherence to general medical therapy [37]. It appears to be a consistent finding that patients with cognitive decline will eventually discontinue—at least temporarily—physical exercise-based therapy, whether due to medical complications or dissatisfaction with the intervention [38]. For this reason, it is essential to assess the extent to which the benefits persist over time.

Addressing these questions is expected to have substantial clinical relevance, as it will enable the development of specific recommendations for institutionalized patients with dementia as well as those cared for at home, thus promoting a comprehensive, long-lasting, and effective approach capable of slowing disease progression, improving established symptoms, increasing independence, reducing caregiver burden, and lowering healthcare costs.

### Objectives

The primary objective of the study was to analyze the impact of a structured and monitored TE program on motor function in patients with dementia in the short and medium term. Secondary objectives included assessing the effectiveness of this protocol on trunk control, balance, gait, and functional independence in patients with MCI and dementia.

## 2. Materials and Methods

### 2.1. Design

An experimental uncontrolled pre–post clinical trial was conducted, approved by the Clinical Research Ethics Committee of the Catholic University of Valencia (Spain) (code UCV/2022-2023/134), and carried out in accordance with the ethical principles of the Declaration of Helsinki. The study was registered as clinical trial ID: ACTRN12624001475538. Additionally, the TREND statement guidelines were followed to ensure the quality of reporting for non-randomized clinical trials.

### 2.2. Participants

The sample size was calculated a priori using G*Power software (version 3.1). For a two-tailed *t*-test for dependent means (matched pairs), considering the Short Physical Performance Battery (SPPB) as the primary outcome, a minimum sample of 35 participants was required to achieve a statistical power of 80% (1-beta = 0.80) and a significance level of 0.05 (alpha = 0.05). This calculation was based on a minimal clinically important difference (MCID) of 1.59 points [39] and a standard deviation of 3.25 (resulting in a Cohen’s effect size dz = 0.49) [40]. To account for an expected 20% attrition rate during the longitudinal follow-up, a total of 44 participants were initially targeted for recruitment.

Participants were recruited from collaborating care centers and patient associations affiliated with the Catholic University of Ávila (Spain) between March and April 2023. Physiotherapists, occupational therapists, social workers, and neuropsychologists from these centers invited eligible patients and users to participate in the project. Subsequently, participants were informed by the research team, and written informed consent was obtained from all participants, family members, or legal guardians prior to data collection.

The inclusion criteria were as follows:

(a) Medical diagnosis of mild-to-moderate cognitive impairment, which was verified through the medical reports of the neurology department responsible for the diagnosis;
(b) Mini-Mental State Examination (MMSE) scores between 10 and 23 (mild–moderate stage). The questionnaire was administered by the neuropsychologist at the social health center;
(c) No regular engagement in physical exercise during the previous 6 months, according to the recommendations set out in the World Health Organization Guidelines on Physical Activity and Sedentary Behavior [41]. This means not having engaged in regular physical activity, not having achieved 150 min of moderate physical activity or 75 min of vigorous physical activity per week, or not having performed muscle-strengthening activities two or more days a week, aerobic exercise for 150 min per week, or multi-component functional balance exercises three or more days a week.

The exclusion criteria were as follows:

(a) Musculoskeletal or cardiovascular comorbidities that limited or contraindicated participation in the exercise protocol, including acute musculoskeletal pathology, non-consolidated fractures, or cardiovascular pathology diagnosed as unstable angina, uncontrolled atrial or ventricular arrhythmias, uncontrolled sinus tachycardia, recent embolism, thrombophlebitis, etc. [42].
(b) Cardiorespiratory sequelae resulting from a SARS-CoV-2 infection or Long COVID, including clinical manifestations such as fatigue, “brain fog” (cognitive impairment), dyspnea, persistent cough, chest pain and muscle aches [43].
(c) Inability to ambulate 10 m independently, requiring assistance from another person or technical aids such as crutches or walkers.

### 2.3. Setting

The sessions were carried out in outpatient centers that confirmed the availability of technical equipment for the exercise program. The physical therapy room had to be equipped with the necessary resources to carry out all types of exercise. These included dumbbells, ankle/wrist weights, or elastic bands, stationary bicycle or treadmill, and mats and rings.

### 2.4. Intervention

Once the eligibility criteria were confirmed and the participants expressed willingness to enroll, a comprehensive baseline assessment (t0) was performed. The 12-week TE intervention then began, with the following characteristics: sessions were given 3 non-consecutive days per week, each lasting 45 min. The program follows the guidelines of the International Association of Geriatrics and Gerontology and combines recommended exercises that are beneficial for cognitive–motor performance [44,45,46,47]. Each week, sessions included endurance, strength, balance and flexibility training. The exercise protocol also incorporated cognitive components aimed at improving executive function, memory, attention, and language abilities. Thus, during motor tasks, participants performed language-related tasks (naming colors, fruits or animals), numerical tasks (counting backwards, and simple addition or subtraction), and similar activities. Sessions were conducted in small groups with a maximum of three patients to ensure individualized attention. In the group sessions, the physical therapist was responsible for creating homogeneous groups in terms of the functional abilities assessed.

At the beginning of the session, a 5 min warm-up was performed, including range of motion exercises, active stretching and walking.

This was followed by 15 min of strength exercises: shoulder flexion and abduction, elbow flexion and extension, squats, hip flexion, extension and abduction, knee flexion and extension, and ankle plantar flexion. Participants were evaluated individually in the first session to determine the appropriate weight, in accordance with the recommendations for working at intensities of 70–85% of 1RM [48,49]. Strength training involved dumbbells, ankle/wrist weights, or elastic bands to target major muscle groups, including 6–8 exercises of 8–10 repetitions each, for 2–3 sets.

The exercise progression was carried out following the 2 × 2 rule, in which when 2 more repetitions than those prescribed for an exercise can be done during 2 consecutive workouts, the load is increased by 2–10% in the next training session.

Endurance training was performed using a stationary bicycle (CXC Matrix Target Training Cycle, Johnson Health Tech, Taichung City, Taiwan) or treadmill (Matrix T70, Johnson Health Tech, Taichung City, Taiwan), for 15 min. The physical therapist was responsible for monitoring the intensity of the exercise through heart rate measured by a chest strap heart rate monitor. In the first session, maximum heart rate was calculated based on age, and an increasing working heart rate was applied in the following sessions, ranging from 60 to 85%, increasing progressively each week according to tolerance. Heart rate monitoring was performed continuously during resistance exercise using Garmin HRM 600^®^ (Garmin Ltd., Olathe, KS, USA) wireless chest strap heart rate monitors, which send real-time heart rate data to the linked device, supervised by the physical therapist. The therapist instructed the patient on the intensity of the exercise so that it was appropriate for the working heart rate range. The participant was encouraged to pedal or walk faster when the working threshold was not reached, while being asked to slow down when the upper range of the maximum working heart rate was exceeded.

**Figure 1:**
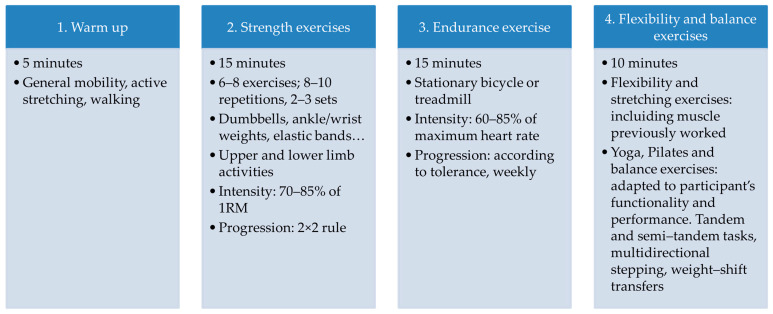
Design of the session and exercises included.

Flexibility and balance exercises included stretching, yoga or Pilates movements were performed at the end of the session for 10 min. Flexibility and stretching exercises included those areas worked during aerobic and resistance exercise, as recommended by the guidelines [50]. Yoga, Pilates and balance exercises were rigorously adapted to each participant according to their level of functionality and performance. They could include tandem and semi-tandem tasks, multidirectional stepping, brisk walking, avoiding obstacles, turns, and weight-shift transfers. The program was continuously adapted to participants’ functional abilities. Design of the session and exercises included are shown in Figure 1.

All study variables were reassessed immediately after the 12-week intervention (t1) and again in a follow-up evaluation 6 months after completion, during which participants had not engaged in any structured or supervised exercise program (t2). All assessments were conducted by members of the research team who were independent from the physiotherapists responsible for delivering and supervising the exercise intervention.

### 2.5. Outcome Measures

Information on gender and age was collected. The primary outcome was general motor function. It was assessed by the SPPB scale, which is a reliable and valid measure of physical performance in adults older than 60 years [51]. It evaluates balance, walking ability and strength of the lower limb, and each test is scored from 0 to 4 points. It provides a total score from 0 (lowest performance) to 12 points (highest performance) [52]. The SPPB scale has been validated in the Spanish older adult population [53].

Secondary outcomes included trunk control, balance, general mobility and gait, and degree of independence in ADL.

Trunk control was assessed by the Trunk Control Test (TCT), which includes rolling to both sides, balance in a sitting position and sitting up from lying down. Each item scores 0, if unable to perform the movement without assistance; 12, if able to perform the movement but in an abnormal style or needing help; or 25, if able to complete the movement normally. The total score (100) is obtained as the sum of the four items [54].

Balance was evaluated by the Berg Balance Scale (BBS). Its 14 items provide a great overview of motor ability and functional task performance. BBS showed excellent test–retest values and an interrater reliability from acceptable in people with AD [55] to excellent in nursing home residents with mild-to-moderate dementia [56]. This test is also useful in predicting the risk of falls, and it can be performed relatively quickly [57].

General mobility, balance and walking ability was assessed by the Timed Up and Go Test (TUGT). The patient sits in the chair with the back against the chair back, and on the command “go”, rises up, walks 3 m, turns, walks back and sits down. This tool is widely used in various populations, and it is validated in AD and dementia patients. TUGT is a reliable outcome measure for people with AD, and it can be used to assess changes in the mobility and walking function [58]. Some recent studies have also highlighted the value of the TUGT performance as a tool to identify cognitive decline and AD [59,60].

The Barthel Index (BI) was used to assess the independence in ADL. This scale is one of the most employed questionnaires in institutionalized and ambulatory patients. The original BI evaluates the ability of feeding, moving from wheelchair to bed and returning, controlling bowels and bladders, doing personal toilet, getting on and off the toilet, bathing, dressing, walking, and ascending and descending stairs. Scores range from 0 (completely dependent) to 100 (completely independent) [61]. Previous studies have confirmed the structural validity, the good reliability, and the ability to detect changes in elderly people in the Spanish version [62].

### 2.6. Data Analysis

Data were analyzed using the SPSS statistical package, version 30.0 (SPSS Inc., Chicago, IL, USA). Descriptive statistics were calculated for all study variables, using frequency distributions for the qualitative variable (gender) and measures of central tendency and dispersion for quantitative variables (age, SPPB, TCT, BBS, TUGT, and BI). Quantitative variables are reported as the mean ± standard deviation, median (interquartile range), and 5th–95th percentile range, as appropriate.

The Shapiro–Wilk test was used to assess the normality of quantitative variables. As none of the outcomes met the assumption of normality at any assessment time point (*p* < 0.05), non-parametric statistical methods were applied for all inferential analyses.

Global longitudinal differences across baseline, post-intervention, and follow-up assessments were examined using the Friedman test. Effect sizes for the Friedman test were quantified using Kendall’s W. When a significant global effect of time was identified, post hoc pairwise comparisons between time points were conducted using the Wilcoxon signed-rank test, with Holm-adjusted *p* values applied to control for inflation of Type I error due to multiple comparisons. Post hoc analyses were performed only for outcomes showing a significant global effect. Statistical significance was set at *p* < 0.05 for all analyses. In addition, data visualization was performed using R, version 4.4.1 (R Core Team, Vienna, Austria). Longitudinal distributions were illustrated using violin plots with embedded summary statistics, generated with the Ggstatsplot package, version 0.13.4 [63], which allows the simultaneous display of data distributions and corresponding non-parametric statistical results.

## 3. Results

Out of a total of 45 subjects recruited, three were excluded at enrollment; *n* = 2 because of the inability to ambulate independently, and *n* = 1 due to severe cognitive impairment (MMSE = 8).

**Figure 2:**
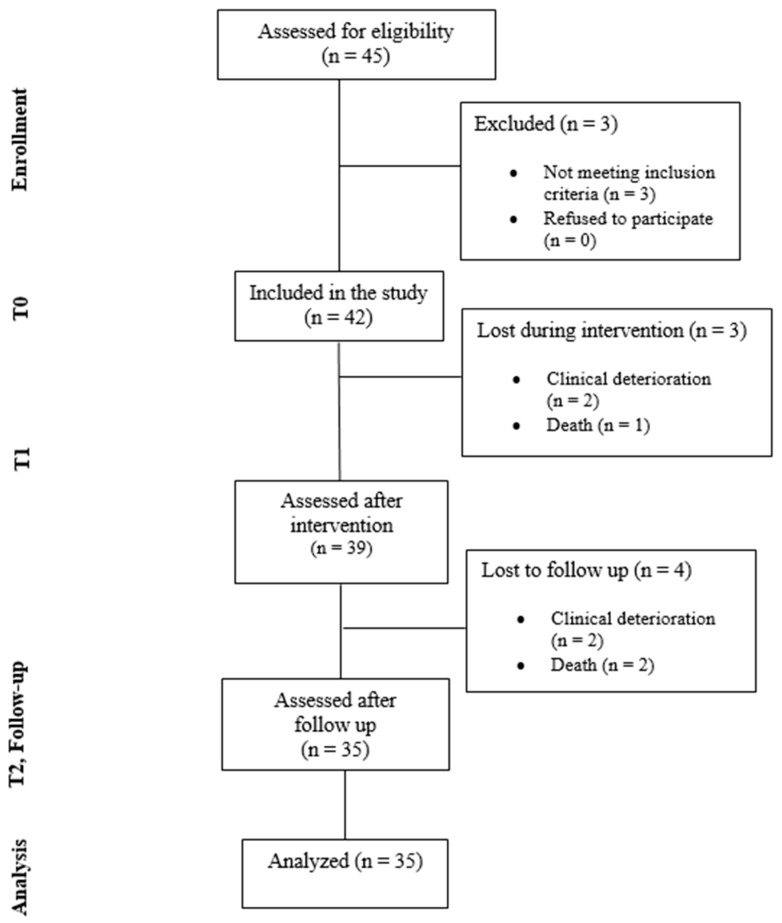
Flow diagram.

During the intervention and follow-up, seven subjects were lost due to clinical worsening and prolonged hospitalization or death. Four people were excluded because of the clinical worsening (two during the intervention protocol and two after completing the protocol) and three due to death (one during the intervention and two after that). Participants who did not complete all study phases from t0 to t2 were excluded from the analysis. A total of 35 participants were finally included (Figure 2). The participants included completed a mean ± SD of 33.70 ± 1.938 sessions, ranging from 29 (80.55% of total sessions) to 36 (100% of total sessions), meaning that all of them met the required 80% attendance rate. No participant was excluded for failing to reach the required level of attendance, suggesting good adherence and acceptance of the proposed protocol.

Of the participants included in the analysis, 13 were men and 22 were women, with a mean age of 68.89 ± 13.43 years. Participant recruitment took place between January and February 2025, the intervention period was conducted between March and May 2025, and the follow-up assessment was carried out between June and December 2025. Table 1 presents the descriptive statistics for the general motor function, trunk control, balance, mobility and gait, and independence in activities of daily living across the three assessment time points (baseline, post-intervention, and follow-up).

Table 1 summarizes the descriptive statistics of the clinical outcomes at baseline, post-intervention, and follow-up. Across all assessment time points, none of the variables met the assumption of normality, as indicated by the Shapiro–Wilk test (all *p* < 0.05). Given the non-normal distribution of the data and the repeated-measures design, non-parametric statistical analyses were subsequently applied to evaluate longitudinal changes.

### 3.1. Global Longitudinal Comparisons

Global longitudinal differences across the three assessment time points were examined using the Friedman test (Table 2). A significant overall effect of time was observed for general motor function (SPPB), χ^2^ = 18.29, *p* < 0.001, indicating a moderate effect, suggesting meaningful changes across the study period. Similarly, balance (BBS) showed a significant global time effect, χ^2^ = 19.21, *p* < 0.001, also with a moderate effect size, reflecting consistent variations in balance performance across assessments.

Trunk control (TCT) exhibited a significant but smaller global effect of time, χ^2^ = 7.95, *p* = 0.019, corresponding to a small effect size, indicating more limited overall changes throughout the follow-up. Likewise, mobility and gait (TUGT) demonstrated a significant global time effect, χ^2^ = 10.11, *p* = 0.006, with a small effect size, suggesting modest but statistically detectable longitudinal differences in functional mobility.

In contrast, no significant global effect of time was detected for independence in activities of daily living (BI), χ^2^ = 4.62, *p* = 0.099. The associated trivial effect size indicates that, at a global level, independence in ADL remained relatively stable across the three assessment points.

**Figure 3:**
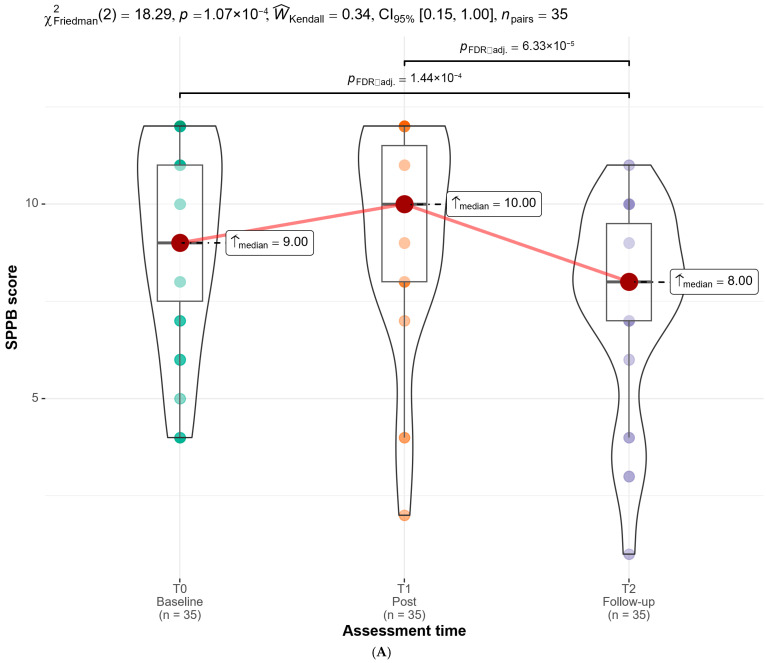

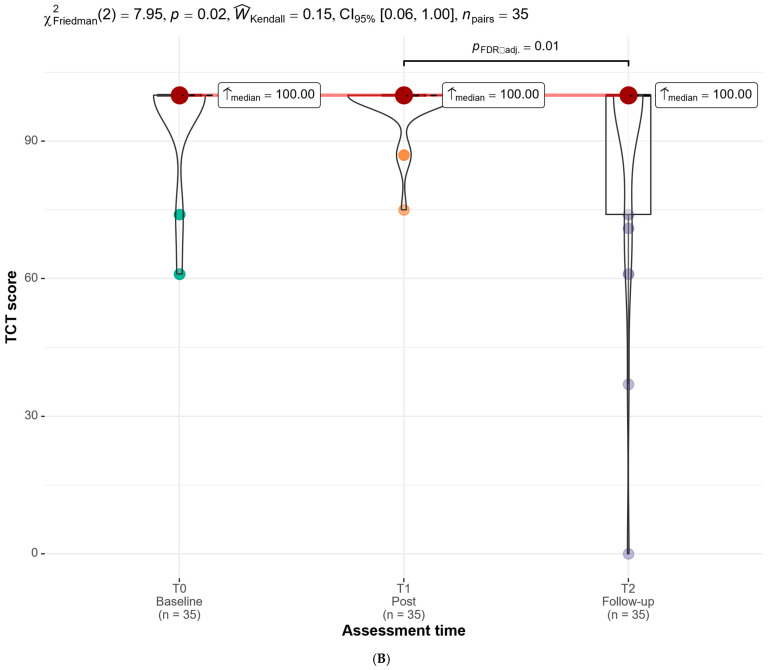

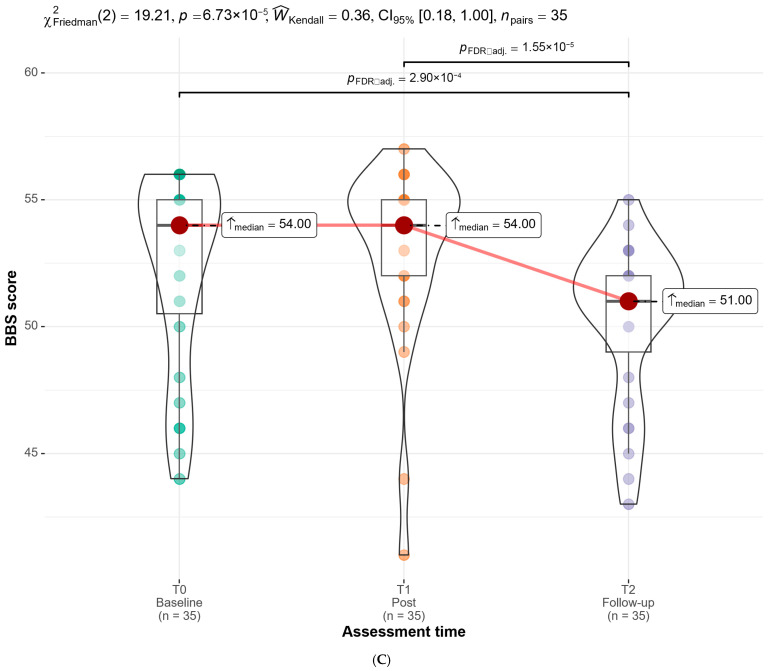

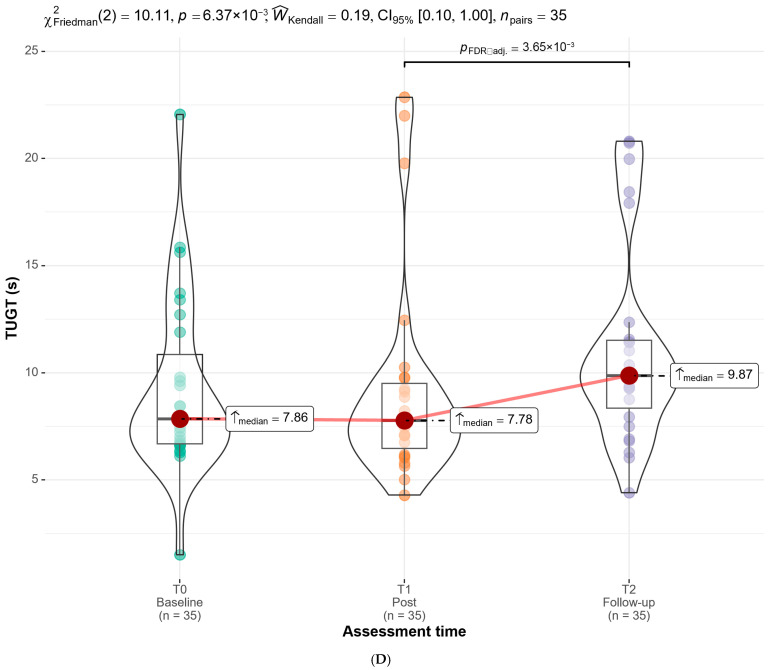

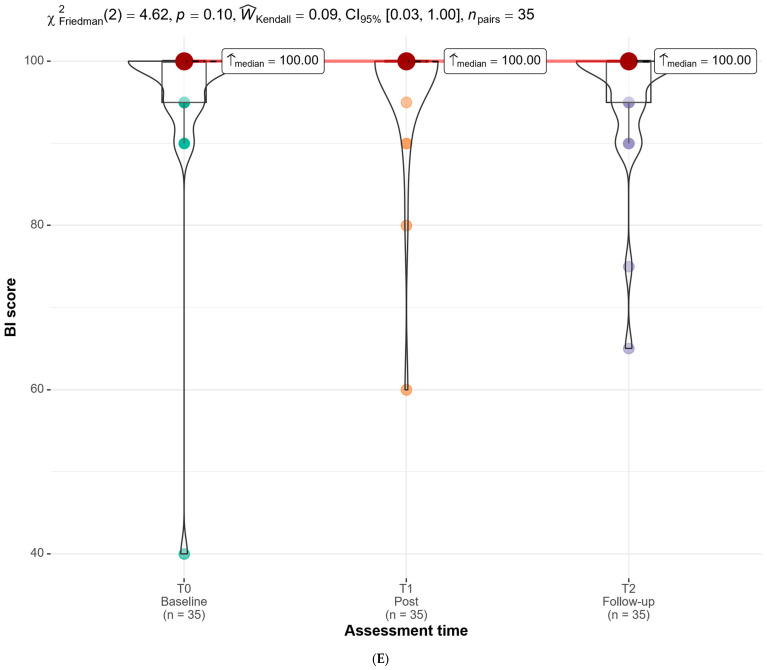
Longitudinal changes in motor function, trunk control, balance, mobility and gait, and independence in activities of daily living from baseline (T0) to post-intervention (T1) and follow-up (T2): (**A**) Short Physical Performance Battery, (**B**) Trunk Control Test, (**C**) Berg Balance Scale, (**D**) Timed Up and Go Test, and (**E**) Barthel Index.

Figure 3 provides a visual representation of the longitudinal trajectories for all functional outcomes across assessment time points. Consistent with the Friedman test results, general motor function (SPPB) and balance (BBS) show clear time-related shifts, particularly at follow-up, whereas trunk control (TCT) and mobility and gait (TUGT) exhibit more modest but detectable longitudinal changes. In contrast, independence in ADL (BI) shows largely overlapping distributions across time points, visually supporting the absence of a significant global effect.

### 3.2. Post Hoc Pairwise Comparisons Between Time Points

Following the identification of significant global effects of time, post hoc pairwise comparisons between assessment time points were conducted using Wilcoxon signed-rank tests (Table 3). To control for the inflation of Type I error due to multiple comparisons, Holm-adjusted *p* values were applied. Post hoc analyses were performed only for outcomes showing a significant global effect in the Friedman test. Accordingly, independence in ADL (BI) was not included in the post hoc analysis, as no significant global time effect was observed for this outcome.

#### 3.2.1. General Motor Function (SPPB)

Post hoc analyses revealed no significant differences between baseline and post-intervention assessments for general motor function (Holm-adjusted *p* = 0.312), despite a large effect size. In contrast, significant differences were observed between baseline and follow-up (Holm-adjusted *p* = 0.010), with a small effect size, as well as between post-intervention and follow-up (Holm-adjusted *p* < 0.001), showing a moderate effect size. These findings suggest that changes in general motor function were more pronounced at follow-up rather than immediately after the intervention.

#### 3.2.2. Trunk Control (TCT)

No significant differences were detected between baseline and post-intervention or between baseline and follow-up assessments for trunk control after adjustment for multiple comparisons (both Holm-adjusted *p* > 0.05). However, a significant difference emerged between post-intervention and follow-up (Holm-adjusted *p* = 0.039), accompanied by a large effect size. This pattern indicates that changes in trunk control were primarily observed during the follow-up period.

#### 3.2.3. Balance (BBS)

Post hoc comparisons showed no significant differences between baseline and post-intervention assessments for balance (Holm-adjusted *p* = 0.217). Significant differences were identified between baseline and follow-up (Holm-adjusted *p* = 0.008), with a small effect size, and between post-intervention and follow-up (Holm-adjusted *p* < 0.001), with a large effect size. These results indicate that balance performance differed mainly at follow-up relative to earlier assessments.

#### 3.2.4. Mobility and Gait (TUGT)

For mobility and gait, no significant differences were found between baseline and post-intervention assessments (Holm-adjusted *p* = 0.160). Significant differences were observed between baseline and follow-up (Holm-adjusted *p* = 0.046), with a moderate effect size, and between post-intervention and follow-up (Holm-adjusted *p* = 0.018), with a large effect size. This suggests that longitudinal changes in functional mobility were evident primarily at follow-up.

### 3.3. Analysis of Results According to the Minimally Clinically Important Difference (MCID)

MCID represents a threshold value of change in patient-reported outcome measure score deemed to have an implication in clinical management, and it is frequently used to interpret the significance of results [64]. However, it is not a constant value of a measurement tool, it can be calculated in many different ways, and it depends on the patient population, diagnosis, stage and severity of the disease [64,65].

Despite this, the results obtained were compared with those of other populations to determine the intensity of the change reflected in each assessment scale. There were no statistically significant differences in independence in ADL between measurements. These changes were also not clinically significant, as it is estimated that the difference should reflect at least 35 points on the BI [66], and the means decreased 0.19 points between T0 and T1, and 1.48 points between T1 and T2.

In contrast, trunk control showed statistically significant differences between measurements; however, in light of the MCID, this change may not be clinically significant, as other studies suggest a variation of 13 points to be significant [67]. However, this has not been specifically studied in patients with dementia.

Regarding general motor function, balance, and gait, this study found a significant worsening between T0 and T2 and between T1 and T2. The literature describes a MCID for the SPPB scale of 1.0 point in older adults [68], and our study reflected a higher variation between the two measurements.

The MCID for the BBS has been studied in several populations, but not in older adults or those with dementia. Available studies report various MCIDs ranging from 2 to 3 points in chronic conditions such as chronic stroke [69] or multiple sclerosis [70] and 6.5 to 11.5 in acute stroke [71] or hip fracture [72], respectively. Our study showed a decrease of 2.04 points between T0 and T2 and 2.74 points between T1 and T2 in the mean BBS score, which could be consistent with an MCID in chronic conditions.

Similarly, the MCIDs for the TUGT report a range of values, between −1.07 and −1.14 s in Parkinson’s disease [73] and −2.82 s for knee osteoarthritis [74]. Our study detected an intermediate variation of −1.76 s between T0 and T2 and −1.83 between T1 and T2.

## 4. Discussion

Protocolized TE performed at moderate intensities in patients with mild-to-moderate cognitive impairment might help maintain—or even slightly improve—functional capacities, while potentially preventing the marked and abrupt physical deterioration associated with both the disease itself and sedentary behavior.

Cognition and functional independence are mutually reinforcing, although in most cases, cognitive decline precedes and predicts functional decline. Therefore, it is essential to promote independence through a comprehensive rehabilitation approach [75,76,77,78].

To our knowledge, this is the first study to describe the deterioration of physical functions in patients with cognitive impairment. Cognitive decline has been described in detail previously [79,80], but the physical aspect has been relegated to the background. Previous studies describe the positive effects of exercise in patients with dementia [81]; however, the degree of permanence of the effects and the natural course of the disease on the physical aspect have not been analyzed.

A systematic review of randomized controlled trials concluded that physical activity can improve both cognitive and non-cognitive variables, although the strength of the evidence ranged from very low to moderate. Most studies on this topic present a high risk of bias and face limitations in measurement instruments, such as floor effects in administered tests [16]. Consistent with these findings, the review by Law et al. concluded that exercise improves global cognitive function, with selective improvements in specific domains—particularly working memory—and more modest effects on language and attention [82]. Wang et al. also pointed that multicomponent exercise has a positive impact on cognitive flexibility, processing speed, verbal fluency and attention in MCI [83].

The present study did not find statistically significant differences following the exercise program. Although the exercise program was designed based on the guidelines and protocols with the best scientific evidence, the duration of the intervention may have been insufficient to trigger the neurobiological cascade that would lead to significant functional changes. Although the intensity of the intervention was adequate and in accordance with the guidelines, future lines of research could study whether a high-intensity program would be capable of achieving significant improvements in functional variables over this period of time.

Another possible reason for not finding differences after the exercise protocol could be related to the participants’ good overall baseline motor function or to the sensitivity of the measurement and psychometric properties of the tests most commonly used in this field, which commonly show a significant ceiling effect.

Similar studies have hypothesized that this may be due to differences in the rate of neurobiological adaptations, which occur more rapidly in individuals with MCI and more slowly in moderate stages of dementia, resulting in non-significant trends in these improvements [81].

Comparable studies confirm that exercise improves mobility and balance, although this may not directly affect fall risk, which might be better prevented through multifactorial interventions [84]. However, other research addressing this issue has not found differences in gait effectiveness between conventional and multimodal interventions [85]. In both cases, the challenge of standardizing the intervention due to individual patient needs complicates the extraction of definitive conclusions [84,85].

Regarding the effectiveness of exercise on gait improvement, other studies report similar findings, where mobility and gait do not improve significantly following an exercise protocol [81,86], but a significant decline is observed in individuals who do not engage in exercise, particularly in TUGT performance and gait cadence [81]. Some studies conclude that physical exercise improves gait speed but does not provide protective effects in the follow-up period for mobility loss [87], consistent with the results of the present study, although other investigations have observed significant improvements after an 8-week follow-up [86].

Other studies have confirmed that exercise programs may delay mobility decline but may not have a significant impact on independence in ADLs [88]. A randomized controlled trial with a large sample of patients reported that a structured exercise program does not produce clinically meaningful benefits in function or quality of life in individuals with dementia or their caregivers [89]; however, it would have been valuable to explore, as in the present study, the progression of these patients after discontinuing the exercise program.

Rist et al. demonstrated that physical activity is associated with a reduced likelihood of developing limitations in instrumental ADLs, which require higher cognitive involvement than basic ADLs [90]. Conversely, physical activity does not appear to protect basic ADLs, suggesting that it provides specific protection against impairments in tasks that are more cognitively demanding [91].

Controversy remains regarding the most appropriate type of exercise or combination thereof, as some studies suggest that interventions such as aerobic exercise or stretching may exert similar effects on cognitive function [92]. However, the effects on motor function have not yet been explored in depth.

A vast body of evidence indicates that aerobic exercise may offer the greatest benefits for these patients, as it significantly increases BDNF levels in the hippocampus and cerebral cortex [18,93], although the mechanisms underlying its stimulation and production remain insufficiently understood [18,94]. Likewise, aerobic exercise is associated with the preservation and enlargement of hippocampal volume in the early stages of dementia [95,96], with the consequent improvement in memory function [96].

Despite the well-documented neurobiological effects triggered by aerobic exercise, evidence suggests that these may not be superior to those achieved through strengthening exercises, which may similarly improve independence, cognitive symptoms, and neuropsychiatric outcomes [44].

Multicomponent exercise includes resistance, strength, balance, and flexibility training and is the most widely recommended approach in older adults [97,98]. Evidence indicates that multicomponent exercise is associated with improvements in global cognitive function [99] and that this effect disappears when the protocol does not include aerobic exercise, as this modality is the one that initiates the neurobiological cascade [97]. Some evidence specifies that resistance exercise is the most beneficial for cognitive function, multicomponent exercise is the most effective to improve executive function, and resistance exercise significantly affects memory function in people with AD [100]. However, a different study points out that aerobic exercise leads to the improvement in cognition, neuropsychiatric symptoms and ADL, resistance exercise also contributes to the previous aspects and enhances muscle strength, but multicomponent exercise may not significantly improve cognitive function [101], neuropsychiatric symptoms and quality of life [102]. Other studies suggest that the most suitable type of exercise to improve cognitive function in MCI may vary depending on the underlying condition: thus, for dementia, resistance training and Tai Chi are effective, for AD, aerobic exercise, and for PD, mind–body exercises [103]. So, the most effective combination, duration, and dosage remain unknown [97].

Recent systematic reviews suggest that physical exercise interventions must be performed two to three times per week [104]. More specifically, evidence points to an optimal frequency of three sessions per week, with one to four sessions improving working memory and more than five providing no additional benefit [105].

The appropriate session duration appears to be 30–60 min [99,104], with 30 min aerobic exercise sessions having a more positive impact on cognitive function than longer ones, likely because excessive fatigue may hinder neuroplasticity. Additionally, the minimum weekly volume of moderate-intensity aerobic exercise should range from 90 to 150 min [105].

The protocol should be carried out for at least two months—and ideally for six—combining aerobic and strengthening exercises to improve cognitive markers as well as physical and neuropsychiatric function [104]. However, this study reveals the importance of maintaining exercise in the long term, as the physical decline in people with dementia is significant within a period of 6 months after cessation of activity. Maintaining moderate physical activity on a continuous basis could slow physical deterioration and promote longer preservation of functional abilities.

There is evidence that the intensity and volume of the intervention are key factors in achieving improvements in physical function [75]. Studies show that gains in gait speed occur only after high-intensity interventions [87], while others suggest that high-intensity programs are capable of slowing physical decline [106]. Although the effects of exercise are never negligible, even at low volume or intensity [23], interventions should adhere to the overload principle [107], although the mechanisms regulating these physiological responses may be altered in patients with dementia [108]. Studies failed to find a relationship between the components of exercise-dosing metrics (intensity, session duration, program length) and BDNF and other plasma neurotrophic biomarkers, possibly due to comorbidities, behavioral and psychological symptoms of dementia, which can affect their exercise participation, and other genetic factors [109].

For cognitive function, studies suggest that the optimal dose may be 650METs-min/week, with aerobic exercise being particularly effective [110]. Integrated physical training programs require 1200 MET-min/week to reach peak effect, whilst resistance training does not yield consistent or significant outcomes, for improving executive function [111]. Nevertheless, several studies highlight the uncertainties about the dose–response relationship [110,111,112]. More studies are needed to compare the effects of interventions based on their intensity, frequency, and duration.

### Limitations

The main limitation of the study is the lack of a control group, which makes it challenging to separate natural disease progression, regression to the mean, seasonal effects, changes in care, etc.

Given the pre–post design without a control group and the sample size, it was not possible to perform adjusted multivariate analyses. However, the use of an intra-subject longitudinal design partially reduced the influence of interindividual variability. It was not possible to create subgroups based on different confounding factors such as age, comorbidities, or physical activity level. A larger sample size could have allowed for subgroup analysis with sufficient statistical power to reach valid conclusions.

There is a ceiling effect for TCT and BI; however, these tests were selected because they are used in centers for the daily assessment of their users and are routinely used to monitor their progress. The participants included, due to the eligibility criteria established, scored high on both tests; however, this ceiling effect is not common in the overall assessment of center users. On the other hand, the use of these tests was accepted because their use in the scientific field is widespread, and this could facilitate the comparison of results with other investigations.

The exercise program was designed based on a combination of guidelines, in the absence of a validated and adequately described protocol for patients with dementia. In all cases, exercises were adapted to the participants’ individual needs, taking into consideration their physical and cognitive abilities, following the guidelines established in the protocol while allowing for some degree of variability in application. The protocol was delivered by three different physiotherapists at their respective clinical sites. To avoid variability in implementation between centers, the following measures were taken: two training sessions were held for the physical therapists responsible for the intervention; specific exercise guidelines were provided, including some model exercises on which to base their own interventions in order to reduce variability in designs [50]; and regular visits were made by research staff to supervise the implementation of the program. No incidents were reported, nor were any interventions modified, as all adhered to the protocol provided by the research team before the start of the intervention.

## 5. Conclusions

Structured exercise training might slightly improve motor function, trunk control, balance and gait in patients with dementia. These potential improvements do not persist over time, and after discontinuing the exercise program, the clinical decline in all functional variables is statistically significant. Exercise training might help slow physical deterioration in patients with mild-to-moderate dementia.

## Figures and Tables

**Table 1:** Descriptive statistics of clinical outcomes across study time points.

Outcome		Mean ± SD	Median (IQR)	5th–95th Range	Normality Shapiro
General motor function (SPPB)	T0	9.07 ± 2.54	9.00 (3.50)	4.30–12.00	0.91 (0.024)
	T1	9.33 ± 2.70	10.00 (3.50)	4.00–12.00	0.86 (0.002)
	T2	7.52 ± 2.55	8.00 (2.50)	3.00–10.00	0.89 (0.007)
Trunk control (TCT)	T0	94.22 ± 12.66	100.00 (0.00)	64.90–100.00	0.50 (<0.001)
	T1	97.63 ± 6.14	100.00 (0.00)	87.00–100.00	0.44 (<0.001)
	T2	87.00 ± 24.40	100.00 (26.00)	44.20–100.00	0.61 (<0.001)
Balance (BBS)	T0	52.26 ± 3.84	54.00 (4.50)	45.30–56.00	0.85 (0.001)
	T1	52.96 ± 3.64	54.00 (3.00)	45.50–56.00	0.79 (<0.001)
	T2	50.22 ± 3.14	51.00 (3.00)	44.30–53.70	0.90 (0.012)
Mobility and gait (TUGT)	T0	9.26 ± 4.13	7.86 (4.16)	6.16–15.78	0.87 (0.003)
	T1	9.19 ± 4.80	7.78 (3.03)	5.21–21.33	0.72 (<0.001)
	T2	11.02 ± 4.58	9.87 (3.15)	6.12–20.50	0.85 (0.001)
Independence in ADL (BI)	T0	95.56 ± 11.63	100.00 (5.00)	90.00–100.00	0.40 (<0.001)
	T1	96.85 ± 8.68	100.00 (0.00)	83.00–100.00	0.43 (<0.001)
	T2	95.37 ± 8.31	100.00 (5.00)	79.50–100.00	0.62 (<0.001)

Note: T0, T1, and T2 correspond to baseline, post-intervention, and follow-up assessments, respectively. None of the outcomes met the assumption of normality at any time point (all *p* < 0.05). Abbreviations: SPPB, Short Physical Performance Battery; TCT, Trunk Control Test; BBS, Berg Balance Scale; TUGT, Timed Up and Go Test; BI, Barthel Index.

**Table 2:** Friedman test results for longitudinal changes in clinical outcomes.

Outcome	Friedman χ^2^ (*p*-Value)	Kendall’s W (Effect Size)
General motor function (SPPB)	18.29 *** (<0.001)	0.339 (moderate)
Trunk control (TCT)	7.95 * (0.019)	0.147 (small)
Balance (BBS)	19.21 *** (<0.001)	0.356 (moderate)
Mobility and gait (TUGT)	10.11 ** (0.006)	0.187 (small)
Independence in ADL (BI)	4.62 (0.099)	0.086 (trivial)

Note. χ^2^ refers to the Friedman chi-square statistic. Kendall’s W is reported as a measure of effect size, with values below 0.10 considered trivial, 0.10–0.29 small, 0.30–0.49 moderate, and ≥0.50 large. * *p* < 0.05; ** *p* < 0.01; *** *p* < 0.001. Abbreviations: SPPB, Short Physical Performance Battery; TCT, Trunk Control Test; BBS, Berg Balance Scale; TUGT, Timed Up and Go Test; BI, Barthel Index.

**Table 3:** Post hoc Wilcoxon signed-rank tests for pairwise comparisons between time points.

Outcome	Comparison	|Z|	*p*-Value	Holm-Adjusted *p*-Value	|r| (Effect Size)
General motor function (SPPB)	T0 vs. T1	3.22	0.312	0.312	0.620 (large)
	T0 vs. T2	0.97	0.005	0.010	0.187 (small)
	T1 vs. T2	2.19	<0.001	<0.001	0.421 (moderate)
Trunk control (TCT)	T0 vs. T1	4.45	0.105	0.210	0.855 (large)
	T0 vs. T2	3.40	0.211	0.211	0.654 (large)
	T1 vs. T2	3.68	0.013	0.039	0.707 (large)
Balance (BBS)	T0 vs. T1	3.00	0.217	0.217	0.578 (large)
	T0 vs. T2	0.63	0.004	0.008	0.120 (small)
	T1 vs. T2	3.30	<0.001	<0.001	0.636 (large)
Mobility and gait (TUGT)	T0 vs. T1	1.42	0.160	0.160	0.273 (small)
	T0 vs. T2	2.28	0.023	0.046	0.439 (moderate)
	T1 vs. T2	2.93	0.006	0.018	0.564 (large)

Note. |Z| indicates the absolute value of the standardized Wilcoxon statistic. Holm-adjusted *p* values were used to determine statistical significance. Effect size is reported as |r|, with values below 0.10 considered trivial, 0.10–0.29 small, 0.30–0.49 moderate, and ≥0.50 large. Abbreviations: SPPB, Short Physical Performance Battery; TCT, Trunk Control Test; BBS, Berg Balance Scale; TUGT, Timed Up and Go Test.

## Data Availability

The data presented in this study are available upon request from the corresponding author. The data are not publicly available due to compliance with data protection regulations.

## Abbreviations

The following abbreviations are used in this manuscript:

AD	Alzheimer’s Disease
ADLs	Activities of Daily Living
BBS	Berg Balance Scale
BI	Barthel Index
MCI	Mild Cognitive Impairment
MCID	Minimally Clinically Important Difference
MMSE	Mini-Mental State Examination
SPPB	Short Physical Performance Battery
TCT	Trunk Control Test
TE	Therapeutic Exercise
TUGT	Timed Up and Go Test
